# Impact of Treviamet® & Treviamet XR® on quality of life besides glycemic control in type 2 DM patients

**DOI:** 10.1186/s12902-023-01492-2

**Published:** 2023-11-08

**Authors:** Asima Khan, Muhammad Adnan Kanpurwala, Riasat Ali Khan, Najum F. Mahmudi, Verumal Lohano, Shakeel Ahmed, Majid Khan, Fareed Uddin, Syed Mohammad Ali, Maliha Saghir, Syed Hussain Baqar Abidi, Jahanzeb Kamal

**Affiliations:** 1 Public Health Department, Baqai Institute of Diabetology and Endocrinology, Karachi, Pakistan; 2 Department of Physiology, Karachi Institute of Medical Sciences affiliated with NUMS, Karachi, Pakistan; 3 Diabetes, Baqai Institute of Diabetology and Endocrinology, Karachi, Pakistan; 4 Primary Care Diabetes Association, Karachi, Pakistan; 5 Diabetes & Endocrinology Department, College of Family Medicine, Karachi, Pakistan; 6 Memon Medical Complex, Karachi, Pakistan; 7 London School of Hygiene and Tropical Medicine, London, UK; 8 Bahria University, Islamabad, Pakistan; 9 Institute of Business Management, Karachi, Pakistan; 10 Medical Education, College of Physicians and Surgeons Pakistan, Karachi, Pakistan

**Keywords:** Quality of life, Type 2 Diabetes Mellitus, Sitagliptin, Metformin

## Abstract

**Background:**

Maintaining the quality of life is the main objective of managing type 2 diabetes (T2DM) (QoL). Since it is a key factor in patient motivation and adherence, treatment-related QoL has always been considered when choosing glucose-lowering medicines. The objective of the study was to evaluate the quality of life besides glycemic control among type 2 diabetes mellitus patients receiving Treviamet® & Treviamet XR® (Sitagliptin with Metformin) in routine care.

**Methods:**

It was a prospective, open-label, non-randomized clinical trial including T2DM patients uncontrolled on Metformin therapy. All patients received Treviamet® & Treviamet XR® for six months. Sequential changes in QoL, fasting plasma glucose, HbA1c, body weight, and blood pressure were monitored from baseline to 3 consecutive follow-up visits. The frequency of adverse events (AEs) was also noted throughout the study.

**Results:**

A total of 504 patients were screened; 188 completed all three follow-ups. The mean QoL score significantly declined from 57.09% at baseline to 33.64% at the 3rd follow-up visit (p < 0.01). Moreover, a significant decline in mean HbA1c and FPG levels was observed from baseline to 3rd follow-up visit (p < 0.01). Minor adverse events were observed, including abdominal discomfort, nausea, flatulence, and indigestion. Gender, HbA1c, diarrhea, and abdominal discomfort were significant predictors of a patient’s QoL, as revealed by the Linear Regression Model (R2 = 0.265, F(16, 99) = 2.231).

**Conclusion:**

Treviamet® & Treviamet XR® significantly improved glycemic control (HbA1c levels) and QoL in T2DM patients without serious adverse events.

**Trial registration:**

ClinicalTrials.gov identifier (NCT05167513), Date of registration: December 22, 2021.

## Introduction

The goal of type 2 diabetes (T2DM) management is to sustain the quality of life (QoL). Many criteria, including gender, age, glycemic control status, disease duration, complications of diabetes, and patient’s physical status, must be considered to achieve these goals. Treatment-related QoL has always been considered significant when selecting glucose-lowering medications since it has been identified as an important component associated with patient motivation and adherence [1].

T2DM is a condition that is intrinsically related to aging, with a dramatically raised prevalence in people getting older. This is mainly a result of insulin secretion deficiencies, insulin resistance (associated with increasing visceral, intramuscular, and intermuscular adiposity), cellular senescence, and lifestyle factors, especially lack of physical activity, typically in the aged population [2, 3]. The number of patients with diabetes over 70 is expected to rise globally over the next three decades due to rising T2DM.

In Pakistan, the quality of life (QoL) of people with diabetes can be influenced by various factors such as socioeconomic status, access to treatment, cultural beliefs, and dietary habits. These factors may differ from other regions and can affect QoL differently. Studies suggest that QoL for diabetes patients in Pakistan [4, 5] is often lower than in other regions [6, 7] due to insufficient education, resources, and a weak healthcare system.

A study reported that 36.5% of the patients with diabetes had not obtained conventional anti-diabetic treatment. This was likely due to the inconvenient usage effects, including limited duration of the activity, insufficient efficacy, and various other side effects, for instance, weight gain, digestive issues, and hypoglycemia [8]. As a result, these medications are considered problematic regarding tolerability and safety. The dipeptidyl peptidase-4 (DPP-4) inhibitor Sitagliptin was approved by the US Food and Drug Administration in 2006. DPP-4 inhibitors are a novel family of anti-diabetic medications that work differently than traditional drugs [9].

Sitagliptin binds to DPP-4 and prevents the breakdown of the glucose-dependent insulinotropic polypeptide (GIP) and glucagon-like peptide-1 (GLP-1). GIP and GLP-1 are both kinds of intestinal incretin hormones that help stimulate insulin secretion while suppressing glucagon secretion [9, 10]. DPP-4 rapidly breaks GIP and GLP-1, depending on the need for glucose in the blood. DPP-4 inhibitors are also linked to a lower incidence of hypoglycemia than traditional glucose-lowering medications. In Pakistan, literature is scarce on the quality of life of patients with diabetes. Hence, the main objective of the study was to evaluate the QoL of type 2 diabetes mellitus patients receiving Treviamet® & Treviamet XR® (Sitagliptin with Metformin) in routine care.

## Methodology

### Trial design

This prospective, open-label, non-randomized clinical trial was designed to evaluate the quality of life (QoL) of type 2 diabetes mellitus patients receiving Treviamet® & Treviamet XR® (Sitagliptin with Metformin) in routine care. The Pakistan Medical Association Committee on Ethics approved the study protocol (Ref No. MN/890/LSQ/12) in accordance with the principles outlined in the Declaration of Helsinki. The trial was registered on clinicaltrials.gov (NCT05167513) prior to participant enrollment. The study continued for 12 months, from June 2021 to May 2022, with each participant enrolling for six months, starting from the initiation of therapy until the final assessment.

### Participants

**Fig. 1:**
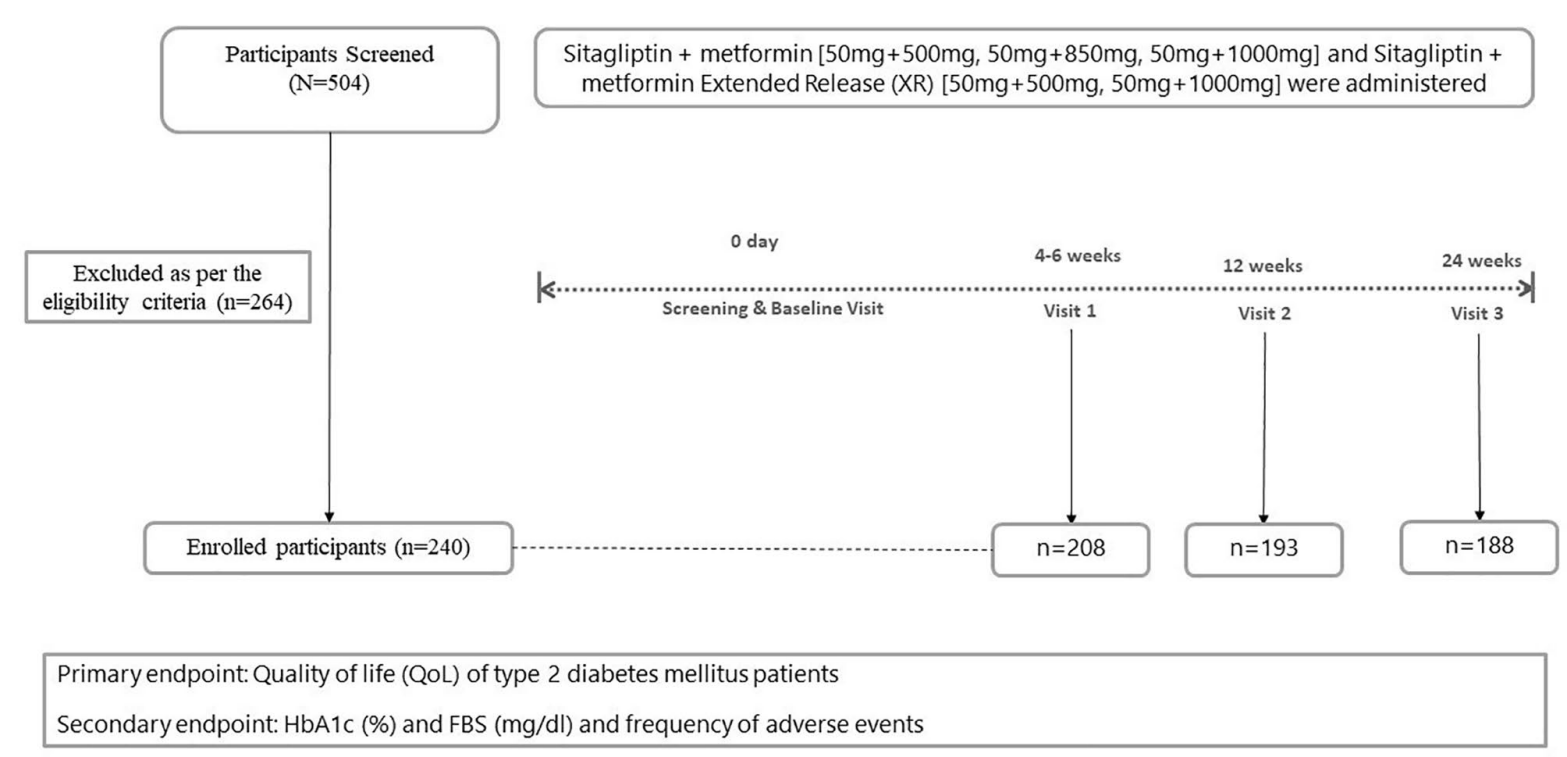
Study flow diagram

The flow diagram demonstrates the flow of participant screening, allocation, and follow-up (Fig. 1). The sample size of 157 was calculated using WHO software “Sample Size Determination in Health Studies,“ considering a mean improvement of 0.7 [8] in quality of life after sitagliptin treatment, 90% power, a 95% confidence interval, and a 5% margin of error based on the expected effect size of the intervention on QoL, the variability of the QoL outcome measure, and the desired level of statistical significance.

Written informed consent was obtained from each patient after providing detailed information regarding the study’s objectives and duration. Of the 504 patients screened, 240 were enrolled initially, 208 showed up on the 1st follow-up visit, 193 on the 2nd, and 188 visited for the 3rd follow-up.

### Assessment of eligibility

A non-probability consecutive sampling method was used to screen patients per study eligibility criteria. The inclusion criteria for the trial were both gender (Pakistan nationals) with T2DM, between ages 18 to 65 years, HbA1c between 7 − 10%, and patients uncontrolled on Metformin and lifestyle modification for at least 3 months. However, patients with type 1 diabetes, pregnant or lactating women, ≥ 1 episode of severe hypoglycemia, diabetic ketoacidosis, and/or hyperosmolar hyperglycemic state in the preceding 3 months, history of pancreatitis or patients with any contraindication including severe renal impairment, hypersensitivity reactions with Sitagliptin or Metformin were excluded from the study.

#### Interventions

Sitagliptin + Metformin [50 mg + 500 mg, 50 mg + 850 mg, 50 mg + 1000 mg] and Sitagliptin + Metformin Extended Release (XR) [50 mg + 500 mg, 50 mg + 1000 mg] were administered to patients who met the eligibility criteria, depending on the patient’s condition for 24 weeks. After recruitment, the patients were invited for three follow-ups, i.e., visit 1 at (4 to 6 weeks), visit 2 at (12 weeks), and visit 3 at (24 weeks) after initiation of therapy.

#### Outcomes

Alongside demographic questions, the first primary endpoint was to evaluate the QoL of type 2 diabetes mellitus patients receiving Treviamet® & Treviamet XR® (Sitagliptin with Metformin)using Diabetes Quality of Life assessment using DQoL-13 interview-based questionnaire [11]. The secondary endpoints included frequency of AEs and change in HbA1c % and FBS (mg/dl) from baseline to the last follow-up visit. Dow Diagnostic Research and Reference Laboratory (DDRRL) was utilized for laboratory testing of diabetes patients.

The DQoL questionnaire is a 13-item scale including three domains satisfaction (6 items), impact (4 items), and worry (3 items). The range of scores for each item is 1 to 5, where 1 denotes never, 2 very seldom, 3 sometimes, 4 often, and 5 denotes all the time [11].

#### Statistical methods

The data were analyzed using SPSS version 22.0. Descriptive statistics were used to present the baseline data, where categorical variables were displayed using frequencies with percentages and continuous variables as mean with standard deviation. Paired Sample T-test was applied to observe the mean change in study variables from baseline to follow-up (for parametric data). At the same time, the Wilcoxon Signed Ranks Test was used to assess the changes in Diabetes Quality of Life (DQoL-13) from baseline to follow-up (Non-parametric data). Multiple linear regression analysis was performed to evaluate the relationship of the DQoL-13 score (dependent variable) with independent confounders like age, gender, weight, duration of diabetes, AEs, HbA1c, and FPG. The effects of Sitagliptin on quality of life, HbA1c levels, and FPG were analyzed using a repeated-measures analysis of variance (ANOVA).

## Results

**Fig. 2:**
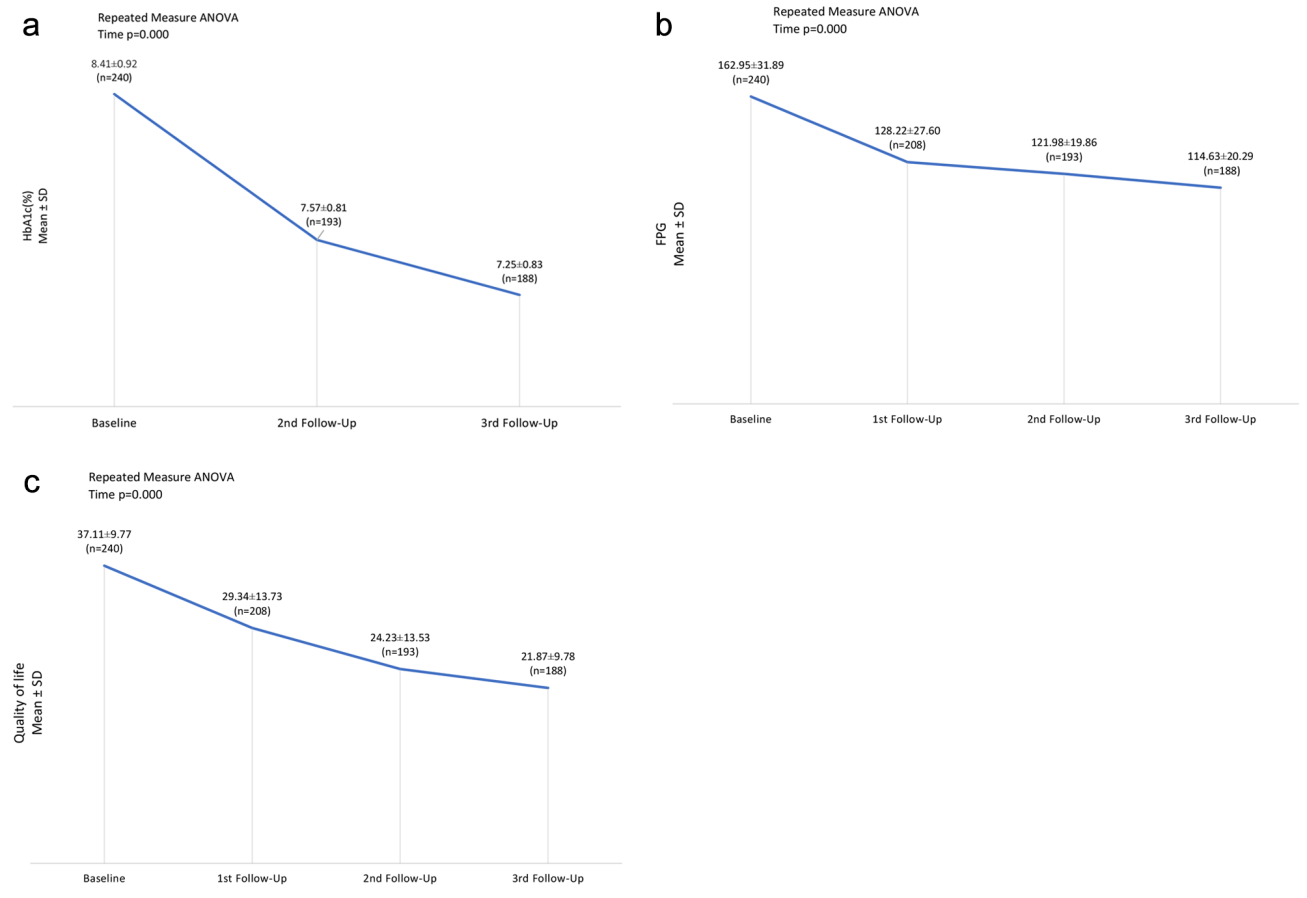
(**a**): HbA1c from baseline to the last follow-up visit; (**b**): Fasting Plasma Glucose from baseline to the last follow-up visit; (**c**): Quality of Life from baseline to the last follow-up visit

A total of 504 patients were screened; 188 completed all three follow-ups. Table 1 shows the baseline characteristics of the study patients. The mean changes in HbA1c, Fasting Plasma Glucose, and Quality of Life measurements of the patients throughout the study period, from the baseline assessment to the follow-up visits, are shown in Fig. 2a, b and c, respectively.

**Table 1:** Baseline characteristics of the study population

Variables				N(%)
**Gender**	Female			122(50.83)
	Male			118(49.17)
**Marital Status**	Married			231(96.25)
	Single			9(3.75)
**Education level**	Graduate or above			83(34.58)
	Secondary or below			128(53.33)
	Uneducated			29(12.08)
**Income group**	Lower-income group (< 19,000/month)			45(18.75)
	Middle income group (19,000–67,000/month)			117(48.75)
	Upper income group (> 67,000/month)			77(32.08)
	Not Reported			1(0.4)
**Number of dependents**	≤ 3			133(55.42)
	4–6			84(35.00)
	7–9			16(6.67)
	≥ 10			7(2.92)
**Smoking** **status**	Current Smoker			14(5.83)
	Ex-smoker (discontinued ≥ 1 year)			8(3.33)
	Never smoked			118(49.17)
	Not Reported			100(41.67)
**Comorbidity**	Hypertension			89(37.08)
	Dyslipidemia			83(34.58)
	Ischemic Heart Disease			9(3.75)
	Chronic Kidney disease			3(1.25)
	Others (Asthma, Gastric issues/ Gout, Thyroid disease)			6(2.50)
**Existing treatment of Metformin**	Dose & Frequency	**500 mg**		163(67.92)
		OD		20(12.27)
		BD		114(69.94)
		TDS		29(17.79)
		**850 mg**		51(21.25)
		OD		4(7.84)
		BD		46(90.20)
		TDS		1(2.00)
		**1000 mg**		26(10.83)
		OD		
		BD		25(96.2)
		TDS		1(3.85)
**Study treatment (Sitagliptin + Metformin)**	Extended-release	50 mg/500 mg	OD	1(16.7)
			BD	5(83.3)
			TDS	
		50 mg/850 mg	OD	
			BD	
			TDS	
		50 mg/1000 mg	OD	2(11.8)
			BD	15(88.2)
			TDS	
	Plain	50 mg/500 mg	OD	13(12.3)
			BD	92(86.8)
			TDS	1(0.9)
		50 mg/850 mg	OD	5(11.6)
			BD	38(88.4)
			TDS	
		50 mg/1000 mg	OD	6(8.8)
			BD	61(89.7)
			TDS	1(1.5)
				**Mean ± SD**
**Age; years**				46.96 ± 9.22
**Weight; kg**				73.84 ± 13.02
**Height; inches**				64.80 ± 3.89
**BMI; kg/m** ^**2**^				27.20 ± 4.65
**Duration of Diabetes; years**				2.41 ± 2.52

Not Reported = Missing data

BD-Twice a day; OD-Once a day; TDS-Thrice a day

The patients’ QoL was significantly improved (p < 0.01), presented in Table 2, i.e., the mean QoL score declined from 57.09% at baseline to 33.64% at the 3rd follow-up visit. Moreover, a significant decline in mean HbA1c (1.24 ± 0.94%, p < 0.001) and FPG (46.63 ± 38.77 mg/dl, p < 0.001) levels were observed from baseline to 3rd follow-up visit, as shown in Table 3.

**Table 2:** Serial changes in quality of life from baseline to the last follow-up visit

Variables		Mean Difference	Z	p-value
**Satisfaction**	Baseline & visit 1	3.78	-9.79	0.000*
	Baseline & visit 2	6.11	-11.76	0.000*
	Baseline & visit 3	7.92	-12.78	0.000*
**Impact**	Baseline & visit 1	2.32	-7.90	0.000*
	Baseline & visit 2	3.95	-10.56	0.000*
	Baseline & visit 3	5.20	-12.04	0.000*
**Worry**	Baseline & visit 1	1.68	-8.24	0.000*
	Baseline & visit 2	2.83	-10.66	0.000*
	Baseline & visit 3	3.70	-12.00	0.000*
**Total**	Baseline & visit 1	7.77	-9.87	0.000*
	Baseline & visit 2	12.89	-11.78	0.000*
	Baseline & visit 3	15.24	-11.62	0.000*

The Wilcoxon Signed Ranks test is applied to observe the mean change in QoL from baseline to follow-up (Non-parametric data). *p < 0.05 is considered statistically significant. Z based on positive ranks

**Table 3:** Serial changes in body weight, blood pressure, FPG, and HbA1c from baseline to the last follow-up visit

Variables			Mean difference		95% CI		p-value
			Mean	SD	LL	UL	
**Weight; kg**		Baseline & visit 1	0.02	3.05	-0.40	0.44	0.933
		Baseline & visit 2	0.85	4.15	0.26	1.45	0.005*
		Baseline & visit 3	1.12	4.18	0.52	1.73	0.000*
**Hypertensive**	**SBP; mmHg**	Baseline & visit 1	11.73	16.61	7.94	15.53	0.000*
		Baseline & visit 2	12.31	16.69	8.36	16.26	0.000*
		Baseline & visit 3	13.25	15.82	9.45	17.05	0.000*
	**DBP; mmHg**	Baseline & visit 1	6.45	11.02	3.93	8.96	0.001*
		Baseline & visit 2	5.25	8.56	3.23	7.28	0.003*
		Baseline & visit 3	6.38	8.83	4.26	8.50	0.000*
**Non-Hypertensive**	**SBP; mmHg**	Baseline & visit 1	1.01	13.07	-1.24	3.26	0.377
		Baseline & visit 2	3.46	12.00	1.31	5.61	0.002
		Baseline & visit 3	3.30	13.05	0.93	5.67	0.007
	**DBP; mmHg**	Baseline & visit 1	-0.02	9.51	-1.65	1.62	0.985
		Baseline & visit 2	0.43	9.36	-1.25	2.10	0.616
		Baseline & visit 3	0.96	10.12	-0.88	2.79	0.304
**FPG; mg/dL**		Baseline & visit 1	34.64	36.80	29.59	39.70	0.000*
		Baseline & visit 2	39.76	33.77	34.95	44.58	0.000*
		Baseline & visit 3	46.63	38.77	41.04	52.23	0.000*
**HbA1c; %**		Baseline & visit 2	0.94	0.71	0.83	1.04	0.000*
		Baseline & visit 3	1.24	0.94	1.11	1.38	0.000*

SBP-Systolic Blood Pressure; DBP-Diastolic Blood Pressure; HbA1c-Glycated Hemoglobin; FPG-Fasting Plasma Glucose; SD-Standard Deviation; LL-Lower Limit; UL-Upper Limit

Paired Sample T-test is applied to observe the mean change in study variables from baseline to follow-up (for parametric data). *p < 0.05 is considered statistically significant

The HbA1c levels were 7 to 9% in 105 by the 3rd follow-up visit. Only 6 patients had an HbA1c level > 9%, and 76 cases had an HbA1c level < 7% by the 3rd follow-up. Minor AEs were observed, including abdominal discomfort 3.8% (n = 9), nausea 3.4% (n = 8), indigestion 3.4% (n = 8), flatulence 2.9% (n = 7), asthenia 2.5% (n = 6), diarrhea 2.5% (n = 6), hypoglycemia 1.7% (n = 4) and vomiting 0.8% (n = 2).

Table 4 shows that gender, HbA1c, diarrhea and abdominal discomfort were significant predictors of a patient’s QoL, as revealed by the Linear Regression Model (R^2^ = 0.265, Adj R^2^ = 0.146, F(16, 99) = 2.231).

**Table 4:** Linear Regression Model to estimate predictors of QoL (last follow-up visit)

Variables	B	SE	β	t	p-value	95% CI	
						LB	UB
Age	0.024	0.101	0.022	0.238	0.813	-0.176	0.224
Gender	5.297	1.998	0.268	2.652	0.009*	1.333	9.260
Marital Status	-4.969	4.380	-0.103	-1.134	0.259	-13.660	3.722
Education level	-1.534	1.333	-0.110	-1.152	0.252	-4.178	1.110
Income	1.011	1.325	0.073	0.763	0.447	-1.617	3.639
Number of dependents	-0.064	0.319	-0.019	-0.201	0.841	-0.697	0.569
Smoking/Tobacco Use	-0.897	1.415	-0.058	-0.634	0.528	-3.704	1.911
Weight^$^	0.097	0.080	0.118	1.208	0.230	-0.062	0.256
Systolic blood pressure^$^	0.127	0.139	0.106	0.913	0.363	-0.149	0.402
Diastolic Blood Pressure^$^	-0.147	0.208	-0.084	-0.705	0.483	-0.560	0.266
Fasting Plasma Glucose^$^	-0.013	0.052	-0.026	-0.258	0.797	-0.117	0.090
HbA1c^$^	3.488	1.101	0.299	3.168	0.002*	1.304	5.673
Hypoglycemia^$^	-16.012	10.305	-0.151	-1.554	0.123	-36.460	4.436
Diarrhea^$^	22.270	9.433	0.416	2.361	0.020*	3.553	40.987
Abdominal discomfort^$^	-21.356	10.853	-0.347	-1.968	0.052*	-42.892	0.179
Indigestion^$^	-9.674	9.694	-0.092	-0.998	0.321	-28.908	9.560

Unstandardized Coefficients (B); Standard Error-SE; Standardized Coefficient-Beta (β); Confidence Interval (CI); Lower Boundary (LB); Upper Boundary (UB)

$At last follow-up visit

R2 = 0.265, Adj R2 = 0.146, F(16, 99) = 2.231, *p < 0.05 is considered significant

Nausea, Vomiting, Flatulence, and Asthenia are constants or have missing correlations; they were deleted from the analysis

No significant difference in DQoL, FPG, and HbA1c levels was observed for the smoking status (p = 0.404; p = 0.780 and p = 0.288, respectively). Similarly, quality of life and HbA1c levels were comparable among male and female patients (p = 0.163 and p = 0.290); only fasting blood glucose varied between genders (p = 0.020).

## Discussion

There is little elucidation regarding treatment-related QoL, specifically in the Pakistani population. Thus, Sitagliptin treatment for glycemic control also improves patient satisfaction, leading to improved QoL, a reasonable hypothesis that is the primary endpoint assessed in the present study. It was observed that after 24 weeks of Sitagliptin medication, the QoL scores improved (p < 0.01). The scores of satisfaction, impact, and worry considerably changed during and after the treatment. Consistent with our findings, Sakamoto et al. demonstrated higher treatment-related QoL beyond glycemic control [8]. Additionally, we also found that individuals with high HbA1c levels were 3.488 times more likely to have a lower quality of life than those with normal or low HbA1c levels (Table 4). In other words, higher HbA1c levels were significantly associated with a greater likelihood of reduced quality of life. Consistently, a sub-analysis of the SPIKE study showed a negative association between QoL score and alterations in the HbA1c level [12].

In this study, it was found that Sitagliptin can reduce levels of HbA1c and FPG. After 12 weeks, there was a reduction of 0.94% and 39.76 mg/dl, respectively. And after 24 weeks, it was 1.24% and 46.63 mg/dl, respectively (Table 3). Similar results were reported in a Korean study by Chung et al., where treatment with Sitagliptin, either alone or combined with Metformin, significantly improved HbA1c and FPG levels [13]. Charbonnel et al. also reported a reduction of 0.65% in HbA1c levels in the Sitagliptin + Metformin group after 24 weeks. However, Hermansen et al. found a higher reduction of 0.74% in HbA1c levels after 24 weeks in the Sitagliptin + Metformin group [14, 15]. Another study in India, which compared the efficacy of Sitagliptin and pioglitazone in combination with Metformin among uncontrolled T2DM patients, reported a significant decrease in mean HbA1c levels in both groups [16]. DPP-4 inhibitors, including Sitagliptin, have also been found to lower blood pressure [17]. A significant reduction in both systolic and diastolic blood pressure was observed in the present study after 24 weeks of Sitagliptin medication (Table 3).

The present study observed that abdominal discomfort, nausea, flatulence, and indigestion were Sitagliptin’s most common AEs. The results of the present study are consistent with previous research conducted by Chawla et al., who reported headache, diarrhea, and nausea as the most common side effects associated with Sitagliptin in a randomized controlled trial [16]. Another study by Liu et al. reported a relatively low incidence of drug-related AEs (4.1%) in patients treated with Sitagliptin and Metformin. These studies suggest that Sitagliptin is generally safe and effective, but more research is needed to compare the safety and efficacy of different DPP-4 inhibitors [18].

There is a dearth of local literature; hence, this study adds local evidence regarding the necessity of monitoring QoL measures and emphasizes the demand for T2DM patients’ QoL-improving therapies. The study also emphasizes how important it is for medical professionals to consider patient QoL factors while managing T2DM patients. However, as mentioned, one limitation of this study is that it was a single-arm study and did not compare the effectiveness of Sitagliptin plus Metformin treatment against a control group. Therefore, it is difficult to determine whether the observed improvements in QoL and glycemic control were solely due to the treatment or could be attributed to other factors. Nevertheless, the results of this study align with previous literature that emphasizes the importance of monitoring multiple parameters beyond just glycemic control in the management of T2DM. The study also highlights the potential benefits of Sitagliptin in improving patients’ QoL measures and glycemic control. In practice, healthcare providers could consider using Sitagliptin as part of a treatment plan for T2DM patients experiencing a decline in their QoL measures. Additionally, larger studies with a control group could provide more robust evidence of Sitagliptin’s effectiveness in improving QoL measures in T2DM patients.

## Conclusion

Study results suggest that Treviamet® & Treviamet XR® (Sitagliptin) is an effective treatment modality for T2DM patients; it significantly improves glycemic control (HbA1c levels) and quality of life without causing any serious adverse events.

## Data Availability

The datasets generated during and/or analyzed during the current study are available in the Mendeley Data repository [Available at Khan, Asima; Kanpurwala, Muhammad Adnan; Khan, Riasat Ali; Mahmudi, Najum F.; Lohano, Verumal; Ahmed, Shakeel; Khan, Majid; Uddin, Fareed; Syed, Muhammad Ali; Saghir, Maliha; Abidi, Syed Hussain Baqar; Kamal, Jahanzeb (2023), “Monitoring Parameters beyond Glycemic Control: Impact of Treviamet® & Treviamet XR® on Quality of Life in Type 2 DM Patients.”, Mendeley Data, V1, doi: 10.17632/6v66krrfts.1]
